# Modulation of Toll-like Receptors with Natural Compounds: A Therapeutic Avenue Against Inflammaging?

**DOI:** 10.3390/ijms262311305

**Published:** 2025-11-22

**Authors:** Corina Andrei, Ciprian Pușcașu, George Mihai Nitulescu, Anca Zanfirescu

**Affiliations:** Faculty of Pharmacy, “Carol Davila” University of Medicine and Pharmacy, Traian Vuia 6, 020956 Bucharest, Romania; corina.andrei@umfcd.ro (C.A.); george.nitulescu@umfcd.ro (G.M.N.); anca.zanfirescu@umfcd.ro (A.Z.)

**Keywords:** toll-like receptors, inflammaging, damage-associated molecular patterns, natural compounds

## Abstract

Chronic low-grade inflammation, or “inflammaging,” is a defining feature of aging and a key driver of functional decline. Among innate immune sensors, Toll-like receptors (TLRs) are central mediators linking cellular stress to sterile inflammation, yet their modulation in physiological aging remains largely overlooked. This review bridges that gap by integrating molecular and clinical evidence on age-associated TLR remodeling and summarizing preclinical data on natural compounds that suppress TLR signaling. Across diverse inflammatory models, phytochemicals such as curcumin, quercetin, resveratrol, baicalin, and glycyrrhizin consistently downregulate Toll-like receptor 2- (TLR2-), Toll-like receptor 4- (TLR4-), and Toll-like receptor 9- (TLR9-) dependent myeloid differentiation primary response 88 (MyD88)/nuclear factor kappa-light-chain-enhancer of activated B cells (NF-κB)/mitogen-activated protein kinase (MAPK) pathways, lowering interleukin-1β (IL-1β), interleukin-6 (IL-6), and tumor necrosis factor- α (TNF-α) while enhancing IL-10. These mechanisms mirror the molecular signature of inflammaging, supporting TLRs as actionable targets for restoring immune balance. Collectively, the evidence positions natural TLR modulators as a promising, yet untapped, avenue for promoting healthy aging and extending healthspan.

## 1. Introduction

Inflammation is a fundamental biological response to tissue injury or infection, essential for defense and repair. However, when unresolved, it transitions from an acute, protective process to a chronic, low-grade inflammatory state that progressively damages tissues and accelerates aging [1]. This persistent, subclinical inflammation, termed “inflammaging”, is characterized by modest but sustained elevations of circulating cytokines such as interleukin-6 (IL-6), tumor necrosis factor alpha (TNF-α), and C-reactive protein (CRP), even in the absence of infection [2]. Unlike acute inflammation, inflammaging is sterile and damage-associated, primarily driven by the accumulation of damage-associated molecular patterns (DAMPs) released from stressed, senescent, or necrotic cells [3].

**Figure 1:**
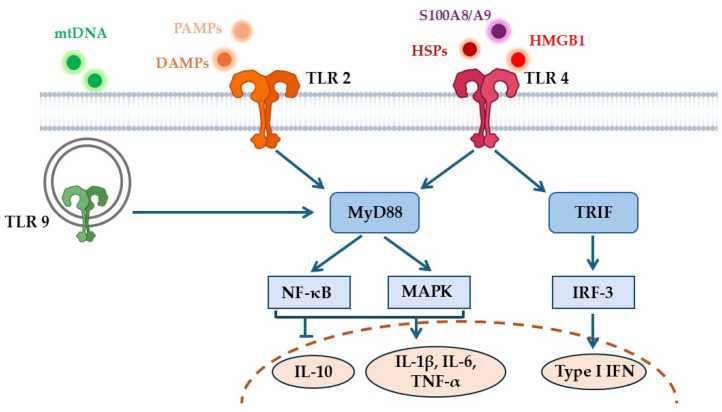
Schematic representation of TLR2, TLR4, and TLR9 signaling pathways. Legend: DAMPs, Damage-associated molecular patterns; HMGBl, High mobility group box 1; HSPs, Heat shock proteins; IFN, interferon; IL, interleukin; MAPK, Mitogen-activated protein kinase; mtDNA, Mitochondrial DNA; MyD88, Myeloid differentiation primary response 88; NF-κB, Nuclear factor kappa-light-chain-enhancer of activated B cells; PAMPs, Pathogen-associated molecular patterns; S100A8/A9, S100 calcium-binding proteins A8 and A9; TLR, Toll-like receptor; TRIF, TIR-domain-containing adapter-inducing interferon-β; TNF-α, Tumor necrosis factor alpha.

Among the molecular mediators of this process, Toll-like receptors (TLRs) occupy a central position. These pattern-recognition receptors bridge cellular senescence, endogenous danger signals, and the inflammatory cascade [4]. TLRs detect not only pathogen-associated molecular patterns (PAMPs) but also endogenous DAMPs that accumulate with aging—such as mitochondrial DNA (mtDNA), advanced glycation end-products (AGEs), high-mobility group box 1 (HMGB1), and extracellular microRNAs (miRNAs) [5,6,7]. Activation of receptors like TLR2, TLR4, and TLR9 by these self-derived ligands triggers chronic NF-κB and interferon signaling (Figure 1), leading to sustained cytokine production and tissue damage [7,8,9].

Thus, TLRs represent a mechanistic intersection between molecular aging (senescence) and chronic innate immune activation. Importantly, TLR expression is not static over the life course. As summarized in Table 1, several TLRs show age-associated changes in expression or signaling capacity in human tissues and circulating immune cells, potentially amplifying the pro-inflammatory milieu characteristic of aging.

These observations support the notion that age-related shifts in TLR dynamics may serve as both biomarkers and mediators of inflammaging. Evidence from human and experimental studies suggests that overactivation of TLR pathways contributes to inflammaging, while their genetic or pharmacologic inhibition can attenuate age-related inflammation and extend health span [16]. Despite increasing recognition of TLRs as central mediators of innate immune activation, the translational potential of TLR modulation for healthy aging remains largely unexplored. Current research has focused mainly on disease models of acute or chronic inflammation, leaving a gap in understanding whether targeting TLR signaling could mitigate age-related inflammatory drift [17,18]. Only a few studies have examined natural or dietary compounds with TLR-inhibitory activity in the context of healthy aging [19,20]. This review addresses this gap by synthesizing evidence on TLR involvement in inflammaging and compiling data on natural compounds that inhibit TLR pathways in inflammatory contexts. Our aim is to identify mechanistically plausible candidates for future anti-inflammaging interventions, bridging molecular immunology with the emerging field of healthspan extension.

## 2. TLRs—Viable Targets for Inflammaging

### 2.1. Genomic and Transcriptomic Evidence in Aging

Accumulating transcriptomic and functional evidence shows that aging profoundly alters innate immune signaling, particularly TLR–mediated pathways [21]. Across multiple human cohorts and cell models, omics analyses consistently reveal shifts in TLR expression or/and downstream responsiveness [10,22]. Aging is characterized by both heightened baseline inflammatory activity and attenuated inducible responses to TLR stimulation [23,24].

The table below (Table 2) summarizes key observational and omics investigations comparing young and elderly populations, highlighting major TLR-related molecular signatures and functional outcomes across tissues and monocyte subsets.

Aging is associated with broad upregulation of pattern-recognition receptor (PRR) genes, most prominently TLR4, reflecting an enhanced innate immune and inflammatory transcriptional program in fibroblasts. Elevated TLR4 expression is linked to cell-cycle gene regulation via TRIF–interferon signaling, while multiple PRRs, including TLR4 and ACE2, are upregulated at the extremes of age, indicating a transcriptional profile primed for heightened innate immune activation [26].

Even within a modest age range (46–68 years), aging is marked by upregulation of innate immune and TLR-related pathways (TLR4, TLR6, TLR9, MyD88, NF-κB), increased expression of oxidative stress and lipid metabolism genes, and reduced mitochondrial and translational activity. Together, these changes define an early transcriptomic signature of immunosenescence and metabolic decline, reflecting systemic immune and metabolic remodeling that underpins low-grade inflammation and reduced physiological resilience [29].

Within immune subsets, classical CD14^+^CD16^−^ monocytes show high baseline expression of TLR2, TLR4, TLR5, TLR6, and TLR8, as well as MyD88, IL-1β, and other NF-κB–related genes, indicating a primed state for TLR-mediated activation compared with CD16^+^ subsets. With age, however, TLR-driven responses weaken: while baseline receptor expression remains stable, monocytes from older adults display reduced transcriptional activation and cytokine production following TLR4 and TLR7/8 stimulation, shifting toward oxidative stress and diminished antiviral signaling. These findings position TLR pathway dysregulation as a central mechanism underlying immunosenescence and inflammaging [27].

### 2.2. Observational Studies: TLR Expression, Inflammation, and Frailty

Correlative studies in human cohorts reinforce the link between TLR activity, systemic inflammation, and age-related frailty. In general, healthy older adults tend to have higher circulating IL-6, TNF-α, and C-reactive protein (CRP) levels than young adults, and those with the highest levels are at greatest risk of morbidity and mortality [30]. TLR upregulation may be one underpinning of this pro-inflammatory milieu [31].

Table 3 summarizes key human observational studies investigating TLR expression and function in relation to frailty, highlighting the study design, analytical methods, TLR targets, and major inflammatory readouts. Together, these findings delineate a pattern of dysregulated TLR signaling—marked by increased basal inflammation, impaired cytokine production, and altered activation phenotypes—that underpins the immune vulnerability of frail older adults.

Aging profoundly reshapes innate immune function, in part through dysregulation of TLR signaling pathways. One key mechanism involves the accumulation of circulating mtDNA, which acts as a persistent endogenous TLR ligand. This continuous stimulation sustains chronic low-grade inflammation, a hallmark of inflammaging, and contributes to frailty and functional decline in older individuals [36].

At the cellular level, aging alters both the composition and activation profile of circulating immune cells. Older adults exhibit an increase in TLR2 expression and in activation markers such as CD86, CD11c, and HLA-DR on monocytes and dendritic cells, alongside a reduction in plasmacytoid dendritic cells (pDCs). In frail individuals, CD40 expression is further elevated on monocytes, reflecting a shift toward a pro-inflammatory phenotype that amplifies basal immune activation and predisposes to chronic inflammation [34].

Aging and frailty are associated with functional impairments in TLR signaling rather than reduced receptor expression. Surface TLR4 levels remained unchanged on monocytes and dendritic cells, but frail older adults showed markedly decreased IL-12p70 and IL-23 production following combined TLR4 and TLR7/8 stimulation. This reduction occurs despite intact proximal signaling pathways (NF-κB, p38, Erk), indicating a downstream defect in cytokine induction. The findings suggest that TLR function is selectively weakened in frailty, leading to diminished Th1/Th17-supporting responses and contributing to the dysregulated innate immunity and immunosenescence characteristic of aging [32].

### 2.3. Endogenous TLR Agonists (DAMPs) Amplifying Inflammaging

The pro-inflammatory milieu of aging arises partly from lifelong exposure to DAMPs released by damaged or senescent cells. Molecules such as mtDNA, HMGB1, S100 proteins, HSPs, and ATP mimic pathogen signals: debris from apoptotic or necrotic cells activates TLRs or the NLRP3 inflammasome, promoting IL-1β and IL-18 secretion. Over time, accumulation of these sterile inflammatory stimuli contributes to immunosenescence and inflammaging [37,38,39]. Senescent cells are a major DAMP source. They release cytokines and alarmins such as HMGB1 and S100 proteins, perpetuating innate immune activation via chronic TLR engagement [40].

#### 2.3.1. mtDNA

mtDNA, which increases in circulation with age, resembles bacterial DNA due to its unmethylated CpG motifs [37]. When released into the cytosol or extracellular space during stress, it activates the endosomal DNA sensor TLR9. TLR9 signals through MyD88 to induce NF-κB–driven inflammatory genes and IRF7-mediated type I interferons [41].

In older adults, elevated mtDNA levels correlate with systemic inflammation. Individuals with sarcopenia showed higher circulating mtDNA and IL-6/IL-8 levels than controls, with mtDNA independently predicting sarcopenia risk [42]. Other studies link mtDNA to elevated TNF-α and IL-6, reinforcing its role as a pro-inflammatory DAMP [43]. Mechanistically, mtDNA–TLR9 interaction in macrophages and dendritic cells triggers NF-κB activation, inducing IL-1β, IL-6, and TNF-α, and can prime the inflammasome [44]. Chronic TLR9 stimulation in aging tissues also upregulates S100A8/A9, creating a feed-forward loop that sustains inflammation and tissue injury [45].

#### 2.3.2. HMGB1 and TLR4

HMGB1, a nuclear chromatin-binding protein, becomes an extracellular alarmin when released by stressed or dying cells. Its levels rise with age and in disorders such as atherosclerosis and neurodegeneration. HMGB1 activates TLR4 and RAGE on immune cells, engaging MyD88/NF-κB and MAPK pathways that drive TNF-α, IL-1β, and IL-6 production [46]. Clinically, elevated HMGB1 correlates with inflammation and frailty. In elderly surgical patients, higher serum HMGB1 was linked to postoperative delirium and increased IL-6 and IL-1β [47]. Chronic HMGB1 elevation also associates with coronary atherosclerosis and frailty, and HMGB1-rich plasma from aged individuals can induce cellular senescence in vitro [48]. Thus, HMGB1 is a central DAMP in inflammaging, sustaining NF-κB signaling and cytokine release through TLR4/MD-2 activation.

#### 2.3.3. S100 Proteins and TLR Signaling

S100A8 and S100A9 form the heterodimer calprotectin, released by neutrophils, monocytes, and senescent cells under stress. Their levels rise with age and in diseases such as atherosclerosis and Alzheimer’s. S100A8/A9 activate TLR4 and RAGE on immune cells, triggering MyD88–NF-κB and MAPK signaling, which induces IL-6 and TNF-α and promotes a pro-inflammatory macrophage phenotype [49].

Elevated S100A8/A9 correlates with inflammation and functional decline in aging tissues. In elderly individuals with periodontal disease—a model of inflammaging—S100A8/A9 expression is TLR-dependent, and TLR blockade reduces both DAMP and cytokine levels. Thus, S100 proteins likely act as endogenous TLR4 ligands amplifying NF-κB–mediated inflammation in aging [50].

#### 2.3.4. HSPs and Other DAMPs

HSPs are intracellular chaperones that, when released extracellularly under stress, act as DAMPs. In older individuals, extracellular HSP70 and HSP90 activate TLR2 and TLR4 on antigen-presenting cells, inducing NF-κB–dependent cytokines. Elevated HSP70 correlates with IL-6 levels and frailty, linking it to age-related inflammation [38].

Extracellular ATP from dying cells activates the P2X7 receptor, triggering the NLRP3 inflammasome and maturation of IL-1β and IL-18. Though ATP does not bind TLRs directly, it amplifies TLR priming and inflammation. Similarly, uric acid—accumulating with age or in gout—activates TLR2/4 and NLRP3, promoting NF-κB activation and IL-1β release. In older adults with chronic kidney disease, uric acid drives vascular inflammation through TLR4–MyD88–NF-κB and NLRP3 pathways [51].

Other endogenous TLR ligands include extracellular DNA/RNA, histones, and oxidized lipoproteins [52]. Oxidized LDL, common in atherosclerosis, activates macrophage TLR4, triggering NF-κB and inflammasome signaling, while self-RNA can engage TLR7/8, contributing to chronic inflammation and autoimmunity in the elderly [53]. Collectively, age-related accumulation of cellular debris (“garb-aging”) chronically engages TLR2/4 (proteins and lipids), TLR9 (mtDNA), and TLR7/8 (RNA), perpetuating innate immune activation.

Thus, DAMP-activated TLRs on macrophages, dendritic cells, and neutrophils trigger signaling cascades converging on NF-κB and AP-1. Most TLRs use MyD88, activating IRAKs and TRAF6. With age, this pathway becomes chronically active: aged immune cells show exaggerated responses despite feedback inhibition [54].

NF-κB drives key features of inflammaging, inducing SASP cytokines (IL-6, IL-1β, TNF-α, CXCL8) and inflammatory enzymes (iNOS, COX-2). Chronic NF-κB activation underlies many age-related pathologies [55,56]. TLR4 can also signal through TRIF to activate IRF3 and type I interferons, which, though antiviral, can become dysregulated in aging and autoimmunity [57].

TLR signaling also primes the inflammasome by upregulating NLRP3 and pro–IL-1β/IL-18, enabling rapid cytokine release upon secondary signals like ATP. Persistent activation induces parainflammation—a low-grade inflammatory state that damages tissues. Over time, continuous stimulation skews monocytes toward a pro-inflammatory state but can also cause TLR tolerance, reducing responsiveness to new challenges [58]. Thus, chronic DAMP–TLR–MyD88–NF-κB signaling sustains IL-6, IL-1β, and TNF-α production, central to the aging immune phenotype.

The persistent cytokine production is linked to age-associated disease. IL-6, a hallmark of inflammaging, predicts morbidity and mortality [59]. TNF-α, another NF-κB target, promotes muscle wasting [60], insulin resistance [61], and neurodegeneration [62]. IL-1β, generated via inflammasome activation, amplifies IL-6 and acute-phase responses; higher IL-1β expression correlates with frailty in older adults [63]. Thus, these inflammatory cascades contribute to sarcopenia, frailty, and multimorbidity.

At the organ level, DAMP-induced TLR signaling drives pathology across systems: TLR4 activation accelerates atherosclerosis, HMGB1 promotes plaque instability, and microglial TLR4 activation by HMGB1 or S100 proteins fuels neuroinflammation and cognitive decline [64,65]. Older adults with pre-existing inflammaging are also more prone to cytokine storms during infection, as seen in COVID-19 [66].

In summary, collective genomic, transcriptomic, and clinical evidence identifies TLRs as viable therapeutic targets in aging. Aging is marked by chronic upregulation of TLR pathways—particularly TLR2, TLR4, and TLR9—driven by persistent stimulation from endogenous DAMPs such as mtDNA, HMGB1, and S100 proteins. This sustained activation of the MyD88–NF-κB axis promotes low-grade inflammation, cytokine overproduction, and tissue dysfunction, hallmarks of inflammaging and frailty. Conversely, impaired inducible TLR responses in older immune cells contribute to immunosenescence and reduced resilience. Targeting TLR signaling therefore addresses both core mechanisms of aging: chronic inflammation and declining immune adaptability.

## 3. Natural Modulators of TLRs

Various plant-derived natural compounds attenuate the inflammatory responses by targeting TLR4-mediated pathways [67,68,69]. Experimental evidence from murine and rat models of metabolic, hepatic, renal, and systemic inflammatory disorders demonstrates that phytochemicals such as curcumin, baicalin, paeoniflorin, quercetin, berberine, and others exert anti-inflammatory effects through inhibition of the TLR4/MyD88/NF-κB cascade and its downstream mediators (e.g., MAPK, AP-1, NLRP3) [70,71,72,73,74,75,76,77].

Table 4 below summarizes key studies reporting ex vivo or in vivo effects of these compounds on TLR4-related signaling and cytokine profiles, detailing the experimental models, administration routes, mechanistic targets, and principal outcomes. Collectively, these findings support the concept that natural bioactive substances can modulate TLR4 signaling, mitigating the excessive inflammatory burden implicated in metabolic dysfunction, liver injury, sepsis, and other inflammation-driven diseases.

Across diverse experimental models, these studies consistently demonstrate that natural compounds act as modulators of TLR4-driven inflammation, converging on a few key molecular events:

- inhibition of canonical TLR4/MyD88/NF-κB signaling. Nearly all compounds (e.g., baicalin, curcumin, berberine, paeoniflorin, quercetin) suppress the MyD88–IRAK–TRAF6 axis, leading to reduced phosphorylation and nuclear translocation of NF-κB p65, and thereby lowering transcription of pro-inflammatory cytokines [70,73,74,77,80].
- downregulation of MAPK and AP-1 pathways. Several agents concurrently inhibit p38, ERK, and JNK activation, attenuating AP-1 (c-Fos/c-Jun)–dependent transcription [68,71,75,76,88].
- suppression of downstream inflammatory mediators. The resulting decreases in TNF-α, IL-1β, IL-6, MCP-1, iNOS, and COX-2 are consistent across models of sepsis, liver injury, kidney damage, and metabolic inflammation [76,81,82,84].
- stabilization of IκB-α and prevention of NF-κB nuclear translocation. This common mechanistic endpoint prevents the persistent transcription of inflammatory genes.
- interference with TLR4–HMGB1 interaction, observed with paeoniflorin, curcumin, and quercetin, highlighting the blockade of DAMP–TLR4 communication as a recurring anti-inflammatory mechanism [75,79,80].
- attenuation of oxidative and fibrotic signaling—many compounds reduce oxidative stress markers (NO, iNOS) and fibrogenic mediators (α-SMA, collagen I), linking TLR4 inhibition to improved tissue remodeling [76,79,81,93].

Several naturally derived compounds suppress inflammation by targeting TLR2-dependent signaling pathways, which are key mediators of sterile inflammation and tissue injury. Evidence from murine and rat models of hepatic, renal, pulmonary, and systemic inflammation indicates that phytochemicals such as curcumin, quercetin, paeoniflorin, resveratrol, berberine, and others inhibit the TLR2/MyD88/NF-κB and MAPK cascades, reducing downstream production of pro-inflammatory cytokines (e.g., TNF-α, IL-1β, IL-6) and oxidative stress markers (Table 5) [74,76,95,97,98,99].

Several natural bioactive compounds have emerged as potent regulators of TLR9-dependent inflammatory signaling, particularly in models involving mitochondrial or nuclear DNA–driven sterile inflammation. Studies in rodent models of sepsis, autoimmune, metabolic, and ischemic injury demonstrate that agents such as curcumin, glycyrrhizin, oxymatrine, tanshinone IIA, oleanolic acid, and gastrodin suppress TLR9/MyD88/NF-κB or related pathways (e.g., IRF5/7, JAK2/STAT3) [101,102,103,104,105,106].

These interventions consistently reduce pro-inflammatory cytokines (TNF-α, IL-1β, IL-6, IL-18), oxidative stress markers (MDA, MPO), and tissue damage indicators (NGAL, KIM-1), while enhancing antioxidant and barrier-protective molecules (e.g., SOD, GSH, zonulin-1, occludin) [101,102,103,104,105,106].

Table 6 summarizes key experimental findings highlighting how TLR9 inhibition by natural compounds mitigates DNA-sensing–driven inflammation, oxidative injury, and immune dysregulation—mechanisms that closely mirror the TLR9–mtDNA axis underlying inflammaging in humans.

To better understand the therapeutic potential of natural Toll-like receptor (TLR) modulators, it is essential to consider their chemical diversity, physicochemical characteristics, and safety profiles. These parameters determine not only the molecular interactions with TLRs but also their pharmacokinetic behavior and potential toxicity in vivo. Table 7 summarizes representative natural compounds with reported TLR-modulating activity, organized by chemical class and key molecular descriptors, along with available safety data from experimental and clinical studies. Presenting this information in an integrated format provides a comparative framework for evaluating structure–activity relationships and for identifying promising candidates for further preclinical or clinical development.

## 4. Discussion

This review addresses a critical gap in current aging research by examining TLRs as mechanistic drivers of inflammaging and as potential therapeutic targets to counteract age-related immune dysregulation. It identifies relevant natural candidates that could be repurposed for anti-inflammaging interventions. The discussion integrates molecular, functional, and translational findings to define how TLR-targeted modulation may restore immune homeostasis and promote resilience in later life.

Older adults exhibit elevated expression of activation markers (CD86, CD11c, HLA-DR) on circulating antigen-presenting cells, higher TLR2 expression on specific monocyte and dendritic cell subsets, and reduced plasmacytoid dendritic cell numbers. Frailty amplifies this primed phenotype through upregulated CD40 expression on monocytes and elevated basal IL-6 levels—defining the pro-inflammatory set point characteristic of inflammaging [29].

Aging profoundly alters innate immune function through dual and seemingly paradoxical trends: baseline activation increases, while inducible responsiveness declines. Older adults display higher expression of activation markers (CD86, CD11c, HLA-DR) on circulating antigen-presenting cells, elevated TLR2 on specific monocyte and dendritic cell subsets, and fewer pDCs. Frailty further amplifies this primed phenotype via upregulated CD40 on monocytes and heightened basal IL-6 levels—defining a pro-inflammatory set-point characteristic of inflammaging [34].

Despite this chronic activation, inducible cytokine responses to TLR ligation are often blunted. In frail elders, TLR4 and TLR7/8 co-stimulation results in reduced IL-12p70 and IL-23 production, despite preserved NF-κB, p38, and Erk activation and unchanged TLR4 surface expression [32]. Multivariate analyses confirm that frailty, dependence, and poor nutrition— rather than chronological age—predict this impairment. Similar patterns of reduced IFN-α, IFN-γ, and IL-1β responses after TLR4/TLR7/8 stimulation indicate weakened Th1/Th17 and antiviral support [32]. In summary, aging manifests as a “primed baseline, smaller fold-change” pattern marked by chronic inflammation but limited adaptability to new immune challenge.

These functional shifts mirror molecular signatures observed in omics studies, which show upregulation of TLR4, TLR6, and TLR9 and their adaptors MYD88 and NFKBIA in older individuals [25,26]. Concomitant elevation of oxidative stress-related genes and diminished mitochondrial activity sustain a feed-forward inflammatory loop. Endogenous DAMPs chronically engage TLRs, activating MyD88/NF-κB signaling and perpetuating IL-6, IL-1β, and TNF-α release.

Thus, TLRs, particularly TLR2, TLR4, and TLR9, represent central molecular hubs linking cellular damage to inflammaging and frailty with TLR4 standing out as a mechanistic bridge between natural anti-inflammatory agents and the molecular hallmarks of inflammaging.

Natural TLR4 modulators may attenuate DAMP-induced TLR4 sustained activation by:

(i) interrupting DAMP–TLR4 feedback loops,
(ii) reducing baseline NF-κB activation and IL-6/TNF output that drive frailty and metabolic decline,
(iii) preserving redox balance to prevent further DAMP release, and
(iv) limiting macrophage and Kupffer cell hyperactivation, which underlies chronic parainflammation in aging tissues [32,33,34,35,36]. Collectively, these mechanisms support TLR4 inhibition as a translational link between nutritional bioactives and the mitigation of age-related inflammatory pathology.

**Figure 2:**
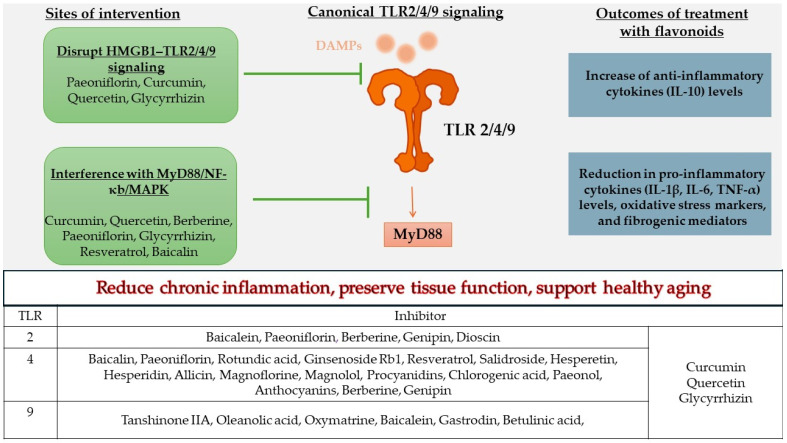
Mechanisms by which flavonoids modulate TLR2/4/9 signaling to reduce chronic inflammation. Legend: DAMPs, Damage-Associated Molecular Patterns; HMGB1, High-Mobility Group Box 1; IL-1β, Interleukin-1 beta; IL-6, Interleukin-6; IL-10, Interleukin-10; MAPK, Mitogen-Activated Protein Kinase; MyD88, Myeloid Differentiation Primary Response 88; NF-κB, Nuclear Factor kappa-light-chain-enhancer of activated B cells; TLR, Toll-Like Receptor; TNF-α, Tumor Necrosis Factor alpha.

Evidence from animal and cell models shows that numerous plant-derived bioactive compounds attenuate TLR-mediated inflammatory signaling. Compounds such as curcumin [70], quercetin [80], berberine [77], paeoniflorin [74], glycyrrhizin [99], resveratrol [98], and baicalin [73] suppress MyD88–IRAK–TRAF6–NF-κB and MAPK pathways, thereby reducing IL-6, TNF-α, and IL-1β production. These agents also limit NLRP3 priming and oxidative stress, restore antioxidant defenses, and preserve mitochondrial homeostasis across models of liver injury, neuroinflammation, metabolic dysfunction, and sepsis [94,98]. Several compounds exhibit multi-TLR modulation, suggesting broader anti-inflammatory capacity in complex age-related contexts. Curcumin, glycyrrhizin and quercetin act on TLR2, TLR4, and TLR9 [70,72,76,80,81,102,103]; berberine and paeoniflorin inhibit both TLR2 and TLR4 [74,95]; and genipin interferes with TLR2/TLR9 signaling [96] (Figure 2). Since multiple DAMPs simultaneously activate distinct TLRs during aging, these pleiotropic modulators may outperform single-target inhibitors by disrupting overlapping inflammatory circuits.

Converging transcriptomic, functional, and preclinical data provides a strong rationale for targeting TLR pathways as a geroprotective strategy. Geriatric syndromes such as frailty, sarcopenia, delirium, and dementia share inflammatory underpinnings, with TLR-mediated innate immune activation emerging as a pivotal mechanistic contributor. Elevated TLR signaling through TLR2, TLR4, and TLR9 correlates with frailty, cognitive impairment, and functional decline. Modulating these pathways could recalibrate the inflammatory set point, reducing basal activation without abolishing inducible defenses—a critical distinction in older adults with immune exhaustion.

Natural multi-TLR modulators, with favorable safety profiles and a history of dietary exposure, represent promising candidates for long-term prophylaxis aimed at extending healthspan rather than merely treating disease.

When evaluating TLR modulators as a means to extend healthspan, several limitations should be noted. First, the current evidence is heavily biased toward preclinical or disease-specific contexts, with aging-specific validation remaining sparse [70,77,102]. Thus, direct data linking these compounds to improved healthspan or reduced frailty in humans are limited. Moreover, substantial variation in dose, formulation, and pharmacokinetics further complicates translation with limited information on long-term safety or bioavailability in older populations [45,63]. Most human studies rely on small, heterogeneous cohorts without consistent control for comorbidities, medications, nutritional status, or sex—factors that shape innate immune tone [109]. Chronological age is often conflated with biological aging or frailty, each of which independently affects TLR function. Furthermore, transcriptomic analyses may also reflect changes in immune cell composition rather than intrinsic pathway activation [35,36].

These discrepancies underscore the need for multi-omic, cell-specific, and protein-level analyses to resolve apparent contradictions between TLR expression and functional responsiveness.

The safety of TLR inhibition in older adults also warrants caution. Because immunosenescence already impairs both innate and adaptive responses, further dampening TLR activity could exacerbate susceptibility to infection or reduce vaccine responsiveness [110]. Clinical experience with anti-inflammatory biologics and TLR4 inhibitors demonstrates increased infection risk, unpredictable immune suppression [111], or unpredictable inflammatory rebounds. Broad immune blockade may fail, or even worsen outcomes, outside defined contexts [112].

Moreover, TLRs are crucial for antitumor immunity and tissue repair; chronic inhibition may impair cancer surveillance or wound healing. Therefore, selective or partial modulation—rather than broad inhibition—should be prioritized. Thus, biomarker-guided interventions in DAMP-dominated states—rather than continuous prophylactic use—may allow inflammatory tone to be lowered without compromising essential defenses. Co-optimization with vaccination is likewise crucial to prevent loss of adjuvant benefit. Clinical studies should stratify by frailty, comorbidities, and polypharmacy and include functional immune readouts (ex vivo TLR responsiveness) alongside clinical endpoints.

Finally, it is important to consider the potential for positivity bias in the literature.

Outcomes often varied based on biological context. TLR modulation is highly context-dependent, governed by dose, formulation, target cell type, ligand, and exposure duration. Reports of natural compounds (e.g., curcumin, quercetin, resveratrol) show that inhibition often occurs only at supra-physiological concentrations or with specialized formulations. Conversely, negligible effects are seen at physiological doses. Some studies reported no effect on TLR expression or cytokine release at very low doses, indicating a narrow therapeutic window for reproducible TLR modulation (Table 8). Variability in purity, vehicle (e.g., DMSO, ethanol), and compound chemistry (e.g., glycosides vs. aglycones) further explains inconsistent outcomes. Some agents display biphasic behavior—low-dose priming followed by inhibition at higher doses—highlighting the need for pharmacokinetic realism and standardized protocols in future studies.

Future research should prioritize:

- Aging-specific translational trials evaluating natural TLR modulators with endpoints such as cytokine profiles (IL-6, IL-1β, TNF-α), circulating DAMPs (mtDNA, HMGB1), and frailty or resilience indices.
- Mechanistic biomarker development, including ex vivo TLR responsiveness, to assess both basal and inducible immune function.
- Combination strategies integrating TLR modulation with DAMP-lowering or senolytic interventions.
- Selective targeting approaches balancing inflammation control with immune competence.

Standardized pharmacokinetic studies, context-specific dosing, and stratification by frailty and comorbidity will be essential to evaluate safety and efficacy. Until such data emerge, natural compounds should be viewed as context-dependent TLR modulators rather than universally effective inhibitors [74,97,98,99].

## 5. Conclusions

In summary, TLR signaling sits at the intersection of innate immune aging, chronic inflammation, and tissue decline. Natural compounds capable of modulating multiple TLR pathways hold promise as prophylactic agents to rebalance the aged systems. By dampening chronic DAMP–TLR–NF-κB activation while preserving adaptive responsiveness, these agents could offer a feasible route to mitigate inflammaging and promote healthy longevity.

## Figures and Tables

**Table 1:** Age-associated alterations in Toll-like receptor (TLR) expression and signaling across different human cell types and tissues.

Body Site/Cell Type (Human)	TLR	Direction with Age	Reference
Peripheral blood—myeloid DCs	TLR1, TLR2, TLR3, TLR8	↓ expression and function	[10]
Peripheral blood—plasmacytoid DCs	TLR9 (and TLR7 responsiveness)	↓ responsiveness/expression signals	[11]
Peripheral blood—Neutrophils	TLR1	↓ surface expression	[12]
Peripheral blood—Monocytes	TLR5	↑ expression and signaling	[13]
Skeletal muscle (vastus lateralis)	TLR4	↑ expression/signaling with aging	[14,15]

Legend: ↓, decreased; ↑, increased; DCs, Dendritic cells; TLR, Toll-like receptor.

**Table 2:** Summary of human omics studies assessing age-related differences in Toll-like receptor (TLR) expression and signaling.

Author (Year)	Sample and Population	Omics Method	TLR-Relevant Findings
Calabria et al. (2016) [25]	Whole blood Healthy adults (46 ± 3 y, N = 11) vs. healthy elderly (68 ± 4 y, N = 9);	Whole-blood microarray + GSEA + qPCR	Enrichment of the Toll pathway gene set with higher expression of TLR4, TLR6, TLR9, MYD88, IKBKG (NEMO), and NFKBIA in the elderly.
Bickler et al. (2021) [26]	Human dermal fibroblast lines spanning ≤ 10 y (N = 14) to ≥ 80 y (N = 33) GSE113957)	Bulk RNA-seq reanalysis	≥80 y vs. ≤10 y groups: Significantly upregulated: TLR3, TLR4, IFIH1 TLR4 showed the strongest effect. Negatively correlated: NOD1, cGAS
Metcalf et al. (2017) [27]	Sorted monocyte subsets Healthy non-frail adults (21–40 y) vs. older adults (≥65 y);	Global transcriptomics after PRR agonists (TLR4, TLR7/8, RIG-I)	Surface TLR expression (TLR3, TLR4, TLR7) did not vary with age. Old vs. young: ↓ TLR-induced cytokine response (IL-1β, IL-6, IFN-γ, TNF-α) ↓ TLR-associated antiviral/interferon genes ↓ NF-κB target genes ↑ Oxidative stress–related transcripts
Wang et al. (2023) [28]	Three monocyte subsets; CD14^+^ monocytes (N = 1202, 44–83 y) Healthy young (19–30 y) vs. elderly (55–86 y);	Bulk transcriptomics + pathway analysis	Old monocytes vs. young monocytes: ↓ IFN-β, IL-1β and IFN-γ response to viral exposure, while ↑ IL-6.

Legend: ↓, decreased; ↑, increased; cGAS, cyclic GMP-AMP synthase; CD14^+^, cluster of differentiation 14 positive; GSEA, gene set enrichment analysis; IFIH1, interferon induced with helicase C domain 1 (MDA5); IFN-β, interferon-beta; IFN-γ, interferon-gamma; IKBKG (NEMO), inhibitor of nuclear factor kappa-B kinase subunit gamma (NF-κB essential modulator); IL-1β, interleukin-1 beta; IL-6, interleukin-6; MYD88, myeloid differentiation primary response 88; NF-κB, nuclear factor kappa-light-chain-enhancer of activated B cells; NFKBIA, nuclear factor kappa-B inhibitor alpha; NOD1, nucleotide-binding oligomerization domain-containing protein 1; PRR, pattern recognition receptor; qPCR, quantitative polymerase chain reaction; RIG-I, retinoic acid–inducible gene I (DDX58); RNA-seq, RNA sequencing; TLR, Toll-like receptor; TNF-α, tumor necrosis factor-alpha.

**Table 3:** Observational studies examining TLR expression, inflammatory responses, and frailty in older adults.

Study (Year)	Settings	Design and Frailty Measure	TLR Target	Main Finding Related to Frailty
Compté et al., 2013 [32]	Belgium; 100 participants aged 23–96 years Subset analysis: 52 participants >75 years old (27 non-frail, 25 frail).	Cross-sectional observational study. Frailty assessed by ISAR scale.	Whole-blood ex vivo; stimulation with LPS (TLR4 ligand) + R848 (TLR7/8 ligand).	Elderly vs. young: TLR4, NF-κB, and p38 expression, and Erk phosphorylation remain stable on monocytes and cDCs ↓ IL-12p70, IL-23 (even more in frail elderly)
Verschoor et al., 2014 [33]	Canada; 129 participants “advanced-age, frail elderly” (61–100 y) vs. young adults (19–59 y)	Cross-sectional observational study. Frailty assessed using the Clinical Frailty Scale (score ≥ 4)	PBMCs—monocytes and DCs; stimulation with Pam3CSK4 (TLR2 agonist) and LPS (TLR4 agonist).	Elderly vs. young: ↑ TLR2 on myeloid DCs ↑ TLR4 on classical monocytes ↓ CCR2 (no change between seniors ↔ frail) ↓ CX3CR1 only in frail elderly
Reitsema et al., 2024 [34]	Netherlands and Sweden; 45 participants Healthy young controls (median age 29 y) vs. healthy (73 y) or frail (76 y) older adults	Observational cross-sectional study. Frailty assessed using the Tilburg Frailty Indicator; Groningen Frailty Indicator, and Fried Frailty Phenotype.	PBMCs—monocyte subsets	Elderly vs. young: ↑ TLR2 on classical monocytes and cDC2 TLR4 ↔ (no significant change) Frailty vs. healthy old age: No significant differences in subset frequencies or other markers (TLR2, TLR4, CD86, CD11c, HLA-DR, PD-L1). Elderly vs. young:
Lukyanova et al. (2024) [35]	Russia 219 nonagenarians (mean age = 92.1 y); control 24 healthy young donors (mean age = 22.5 y).	Observational cross-sectional study nonagenarians categorized as with/without frailty and as successful vs. pathological aging based on presence of four geriatric syndromes (frailty, dementia, sarcopenia, reduced functional activity).	TLR2 gene expression assessed in peripheral blood leukocytes.	↑ IL1β and TLR2 expression ↓ IL10 expression Frailty vs. non-frailty: ↑ IL1β and TLR2 expression Successful vs. pathological aging: ↑ IL10 expression Similar IL1β and TLR2 levels of expression
Nidadavolu et al. (2023) [36]	USA; community-dwelling older adults (N = 672; mean age 80.4 ± 7.2 y)	Observational, cross-sectional and longitudinal analyses. Frailty measured as a composite *z*-score including grip strength, timed walk, body mass index, and fatigue	Indirect innate immune activation assessed via circulating cell-free mtDNA	Elevated mtDNA levels correlated with ↑ frailty, ↓ gait speed, ↓ grip strength, ↓ cognitive performance, and ↑ mortality risk. cf-mtDNA-induced activation of TLR9: ↑ CRP, ↑ TNF-α, and ↑ IL-6

Legend: ↓, decreased; ↑, increased; ↔, unchanged; ACE2, angiotensin-converting enzyme 2; CCR2, C-C chemokine receptor type 2; cDC, conventional dendritic cell; cDC2, conventional dendritic cell type 2; cf-mtDNA, circulating cell-free mitochondrial DNA; CRP, C-reactive protein; CX3CR1, C-X3-C motif chemokine receptor 1; DC, dendritic cell; Erk, extracellular signal-regulated kinase; HLA-DR, human leukocyte antigen—DR isotype; IL-1β, interleukin-1 beta; IL-6, interleukin-6; IL-10, interleukin-10; IL-12p70, interleukin-12 p70 subunit; IL-23, interleukin-23; ISAR, Identification of Seniors at Risk; LPS, lipopolysaccharide; mtDNA, mitochondrial DNA; NF-κB, nuclear factor kappa-light-chain-enhancer of activated B cells; PBMC, peripheral blood mononuclear cell; PD-L1, programmed death-ligand 1; p38, p38 mitogen-activated protein kinase; R848, resiquimod; TLR, Toll-like receptor; TNF-α, tumor necrosis factor-alpha.

**Table 4:** Natural compounds targeting TLR4 signaling pathways in experimental models of inflammation and tissue injury.

Natural Compound	Sources	Experimental Model	Mode of Administration	TLR4-Related Mechanism	Main Outcomes	Reference
Baicalin	*Scutellaria baicalensis Georgi*	MCD-induced NAFLD C57BL/6J mice	50 mg/kg, orally, 4 weeks	TLR4/MyD88/NF-κB/p38 MAPK inhibition	↓ TNF-α ↓ IL-1β ↓ IL-6 ↓ CCL2 ↓ CXCL2 ↓ ICAM ↓ VCAM ↓ ELAM	[73]
Ginger essential oil	*Zingiber officinale*	P-HFD/LPS-induced NASH C57BL/6 J mice	12.5–125 mg/kg, orally, 12 weeks	TLR4/NF-κB inhibition	↓ TNF-α ↓ IL-1β ↓ IL-6	[67]
Paeoniflorin	*Paeonia lactiflora Pall*	Hepatic ischemia/reperfusion injury C57BL/6 mice	100 mg/kg, orally, 3 times (every 8 h) before surgery	TLR4/HMGB1/ERK1/2/JNK1/2/p38/NF-κB inhibition	↓ TNF-α ↓ IL-1β	[75]
		Type 2 diabetes-induced nephropathy db/db mice	15–60 mg, i.p., 2 weeks	TLR4/MyD88/IRAK1/NF-κB/iNOS inhibition	↓ TNF-α ↓ IL-1β ↓ MCP-1 ↓ CD68^+^	[74]
Rotundic acid	*Ilex rotunda*	HFD-induced NASH C57BL/6 mice	30 mg/kg, orally, 3 weeks	TLR4/MyD88/MAP3K8/MAPK3/AP-1 (c-Fos/c-Jun) inhibition	-	[68]
Ginsenoside Rb1	*Panax ginseng*	D-GalN/LPS-induced acute liver injury C57BL/6 mice	30–60 mg/kg, i.p. 3 days before D-GalN/LPS treatment	TLR4/MyD88/NF-κB/NLRP3 inhibition	↓ TNF-α ↓ IL-6 ↓ IL-1β ↓ IL-18	[69]
Curcumin	*Curcuma longa Linn*	Concanavalin A–induced autoimmune hepatitis BALB/c mice	200 mg/kg, orally, 40 min before Concanavalin A injection	TLR4 inhibition	↓ TNF-α ↓ IFN-γ ↑ IL-10 ↓ Kupffer cell infiltration (F4/80^+^)	[72]
		LPS/DCL-induced hepatotoxicity Wistar rats	200 mg/kg, orally, for 7 days before LPS/DCL and twice (2 h and 8 h) after LPS/DCL	TLR4/NF-κB/MAPK (p38/JNK) inhibition	↓ TNF-α ↓ IL-6 ↓ NO	[78]
		Traumatic brain injury C57BL/6 mice	50–200 mg/kg, i.p., single dose	TLR4/MyD88/NF-κB inhibition	↓ IL-1β ↓ IL-6 ↓ TNF-α ↓ MCP-1 ↓ RANTES	[70]
		CCl_4_-induced hepatic fibrosis Sprague-Dawley rats	200 mg/kg, orally, 6 weeks	TLR4/HMGB1 inhibition	↓ col3a1 ↓ alpha-SMA ↓ TNF-a ↓ IL-6 ↓ MCP-1	[79]
Triazole curcumin	*Curcuma longa* Linn	LPS-induced ALI Kunming mice	2.5–20 mg/kg, orally, single dose, before LPS treatment	TLR4/MyD88/NF-κB/AP-1 inhibition	↓ TNF-α ↓ IL-6 ↓ fibrosis	[71]
Quercetin	*Allium cepa* *Vitis vinifera* *Solanum lycopersicum* *Brassica oleracea*	CCl_4_-induced hepatic inflammation ICR mice	40 -80 mg/kg, orally, 1 week	TLR4/NF-κB (p65)/MAPK (p38/JNK/ERK) inhibition	↓ iNOS ↓ IL-1β ↓ COX-2 ↓ NO	[76]
		Concanavalin A– induced hepatitis BALB/c mice	50 mg/kg, i.p., single dose before concanavalin A	TLR2/HMGB1/NF-κB inhibition	↓ TNF-α ↓ Interferon-γ ↓ IL-4	[80]
Glycyrrhizin	*Glycyrrhiza glabra*	LPS-induced ALI BALB/c mice	50 mg/kg, i.v., immediately and 12 h following LPS injection	TLR4/NF-κB inhibition	↓ TNF-α ↓ IL-1α ↓ IL-6 ↓ neutrophil and macrophage infiltration ↓ MPO ↓ COX-2 ↓ iNOS	[81]
Resveratrol	*Vitis vinifera*	LPS-induced ALI BALB/c mice	5–45 mg/kg, orally, 3 days before LPS injection	TLR4/MyD88/NF-κB inhibition	↓ IL-6 ↓ COX-2	[82]
Salidroside	*Rhodiola rosea*	LPS-induced ALI Sprague–Dawley rats	20–40 mg/kg, orally, 3 days	TLR4/NF-κB inhibition	↓ TNF-α ↓ IL-6 ↓ IL-1β	[83]
Hesperetin	*Citrus* spp.	LPS-induced ALI C57BL/6 mice	10–30 mg/kg, orally, single dose	TLR4/MyD88/TRAF6/TAK1/NF-κB (p65) inhibition; IκB-α stabilization	↓ TNF-α ↓ IL-6 ↓ NO	[84]
Hesperidin	*Citrus* spp.	CLP-induced sepsis-associated lung injury albino mice	10–20 mg/kg, orally, single dose	Hsp70/TLR4/MyD88 inhibition	↓ TNF-α ↓ IL-6 ↓ IL-1	[85]
Allicin	*Allium sativum*	LPS-induced ALI Sprague-Dawley rats	25–100 μg/mL, i.p., every 12 h, for 24 h	TLR4/MyD88/NF-κB inhibition	↓ TNF-α ↓ IL-6 ↓ IL-1β	[86]
		Alcohol-induced hepatic steatosis C57BL/6 mice	5–20 mg/kg, orally, 4 weeks	TLR4/CD14 inhibition	↓ TNF-α ↓ IL-1β ↓ IL-6	[87]
Magnoflorine	*Magnolia* spp. *Aristolochia* spp.	LPS-induced ALI BALB/c mice	5–20 mg/kg, i.p., three times at 0, 8, 16 h after LPS injection	TLR4/NF-κB/MAPK (p38/ERK/JNK) inhibition; IκBα stabilization	↓ TNF-α ↓ IL-1β ↓ IL-6	[88]
Magnolol	*Magnolia officinalis*	LPS-induced ALI BALB/c mice	5–20 mg/kg, i.p., single dose	TLR4/NF-κB inhibition; IκBα stabilization	↓ TNF-α ↓ IL-1β ↓ IL-6	[89]
Procyanidin	*Vitis vinifera*	TiO_2_ nanoparticle–induced hepatotoxicity Albino rats	75 mg/kg, orally, 30 days	TLR4/NIK/NF-κB inhibition	↓ TNF-α	[90]
Chlorogenic acid	*Camellia sinensis* *Meum athamanticum*	LPS-induced AKI C57BL/6 mice	5–20 mg/kg, i.p., single dose, 1h before LPS injection	TLR4/MyD88 inhibition; NF-κB (p65) inhibition; IκB-α stabilization	↓ TNF-α ↓ IL-1β ↓ IL-6	[91]
Paeonol	*Paeonia moutan Sims*	LPS-induced AKI BALB/c mice	12.5–50 mg/kg, orally, 7 days	TLR4/IKKβ/NF-κB (p65) inhibition; IκBα stabilization	↓ TNF-α ↓ IL-1β ↓ IL-6	[92]
Anthocyanins	*Myrica rubra*	Cerebral ischemia–reperfusion injury ICR mice	100–300 mg/kg, orally, 1 week	TLR4/NLRP3 inhibition	↓ TNF-α ↓ IL-18 ↓ caspase-1 ↓ NO	[93]
Berberine	*Coptis chinensis*	CLP-induced sepsis Wistar rats	25–50 mg/kg, orally, 5 days	TLR4/NF-κB/NLRP3 inhibition	↓ TNF-α ↓ IL-1β	[94]
		LPS-induced sepsis-associated myocardial injury Sprague Dawley rats	50 mg/kg, orally, 3 days before LPS injection	TLR4/NF-κB (p65) inhibition	↓ TNF-α ↓ IL-1β	[77]
		Acute alcohol–induced gastrointestinal injury ICR mice	75–300 mg/kg, orally, 1h before alcohol administration	TLR4 inhibition	↓ TNF-α ↓ IL-1β	[95]
Genipin	*Gardenia jasminoides*	CLP–induced sepsis ICR mice	1–5 mg/kg, i.v., immediately (0 h) or 0 and 24 h after CLP	TLR2/HMGB1/MyD88/TRIF/NF-κB (p65)/MAPK (p38/JNK/ERK)/IRF3; IκBα stabilization	↓ TNF-α ↓ IL-1β ↓ IL-6	[96]

Legend: ↓, decreased; ↑, increased; ALI, acute lung injury; alpha-SMA, alpha-smooth muscle actin; AKI, acute kidney injury; AP-1, activator protein-1; CCl_4_, carbon tetrachloride; CCL2, C-C motif chemokine ligand 2; CLP, cecal ligation and puncture; col3a1, type III collagen a 1; COX-2, cyclooxygenase-2; CXCL2, C-X-C motif chemokine ligand 2; D-GalN, D-galactosamine; DCL, diclofenac; ELAM, endothelial-leukocyte adhesion molecule; ERK1/2, extracellular signal-regulated kinase ^1^/_2_; F4/80^+^, macrophage marker F4/80-positive cells; HFD, high-fat diet; HMGB1, high mobility group box 1; Hsp70, heat shock protein 70; ICAM, intercellular adhesion molecule; ICR, Institute of Cancer Research (mouse strain); IFN-γ, interferon gamma; IL-1α, interleukin-1 alpha; IL-1β, interleukin-1 beta; IL-4, interleukin-4; IL-6, interleukin-6; IL-10, interleukin-10; IL-18, interleukin-18; iNOS, inducible nitric oxide synthase; IκB-α, inhibitor of nuclear factor kappa B alpha; IRAK1, interleukin-1 receptor-associated kinase 1; IRF3, interferon regulatory factor; JNK, c-Jun N-terminal kinase; MAP3K8, mitogen-activated protein kinase kinase kinase 8; MAPK, mitogen-activated protein kinase; MCD, methionine- and choline-deficient; MCP-1, monocyte chemoattractant protein-1; MPO, myeloperoxidase; MyD88, myeloid differentiation primary response 88; NAFLD, non-alcoholic fatty liver disease; NASH, non-alcoholic steatohepatitis; NF-κB, nuclear factor kappa B; NIK, NF-κB-inducing kinase; NLRP3, NOD-, LRR- and pyrin domain-containing protein 3; NO, nitric oxide; p38 MAPK, p38 mitogen-activated protein kinase; LPS, lipopolysaccharide; MCP-1, monocyte chemotactic protein 1; P-HFD, processed high-fat diet; RANTES, regulated on activation, normal T cell expressed and secreted; TAK1, transforming growth factor beta-activated kinase 1; TLR4, toll-like receptor 4; TNF-α, tumor necrosis factor alpha; TRAF6, TNF receptor-associated factor 6; TRIF, TIR-domain-containing adapter-inducing interferon-β; VCAM, vascular cell adhesion molecule.

**Table 5:** Natural compounds targeting TLR2 signaling pathways in experimental models of inflammation and organ injury.

Natural Compound	Sources	Experimental Model	Method of Administration	TLR2-Related Mechanism	Main Outcomes	Reference
Curcumin	*Curcuma longa Linn*	Concanavalin A–induced autoimmune hepatitis BALB/c mice	200 mg/kg, orally, 40 min before Concanavalin A injection	TLR2 inhibition	↓ TNF-α ↓ IFN-γ ↑ IL-10 ↓ Kupffer cell infiltration (F4/80^+^)	[72]
		CCl_4_-induced hepatic fibrosis Sprague-Dawley rats	200 mg/kg, orally, 6 weeks	TLR2/HMGB1 inhibition	↓ col3a1 ↓ alpha-SMA ↓ TNF-a ↓ IL-6 ↓ MCP-1	[79]
Glycyrrhizin	*Glycyrrhiza glabra*	Lung ischemia–reperfusion injury BALB/c mice	200 mg/kg, i.p., single dose	TLR2/MyD88/NF-κB inhibition	↓ IL-1β ↓ IL-6	[99]
Quercetin	*Allium cepa* *Vitis vinifera* *Solanum lycopersicum* *Brassica oleracea*	CCl_4_-induced hepatic inflammation ICR mice	40–80 mg/kg, orally, 1 week	TLR2/NF-κB (p65)/MAPK (p38/JNK/ERK) inhibition	↓ iNOS ↓ IL-1β ↓ COX-2 ↓ NO	[76]
		Concanavalin A-induced hepatitis BALB/c mice	50 mg/kg, i.p., single dose before concanavalin A	TLR2/HMGB1/NF-κB (p65) inhibition; IκB stabilization	↓ TNF-α ↓ Interferon-γ ↓ IL-4	[80]
Quercetin/Baicalein	Quercetin: *Allium cepa* *Vitis vinifera* *Solanum lycopersicum* *Brassica oleracea* Baicalein: *Lepisorus ussuriensis* *Scutellaria prostrata*	MCT-induced SOS Sprague Dawley rats	40 mg/kg, intragastrical administration, twice at 6 h and 30 h after MCT administration	TLR2/MyD88/NF-κB (p-p65/nuclear-p65/p-IκB)/Egr1/MAPK (p-ASK1/p-MEK1/2/p-cRaf/p-MKK3/6/p-MKK4/p38/JNK/ERK 1/2)/PI3K/AKT/mTOR inhibition; Nrf2 activation	↓ TNF-α ↓ IL-1β ↓ MDA ↓ MPO ↓ MMP-9 ↓ Serpine1 ↓ TF ↑ GCLC ↑ GCLM	[100]
Paeoniflorin	*Paeonia lactiflora Pall*	Type 2 diabetes-induced nephropathy db/db mice	15–60 mg, i.p., 2 weeks	TLR2/MyD88/IRAK1/NF-κB/iNOS inhibition	↓ TNF-α ↓ IL-1β ↓ MCP-1 ↓ CD68^+^	[74]
Berberine	*Coptis chinensis*	Acute alcohol–induced gastrointestinal injury ICR mice	75–300 mg/kg, orally, 1h before alcohol administration	TLR2 inhibition	↓ TNF-α ↓ IL-1β	[95]
Resveratrol	*Vitis vinifera*	CCl_4_–induced liver fibrosis BALB/c mice	400 mg/kg.d, i.p., 5 weeks	4 weeks: TLR2/MyD88/ERK/NF-κB (p50)/NLRP3 activation 5 weeks: TLR2/MyD88/ERK/NF-κB (p50)/NLRP3 inhibition	4 weeks: ↑ IL-10 ↑ Caspase-1 ↑ IL-1β ↑ IL-18 5 weeks: ↓ IL-10 ↓ Caspase-1 ↓ IL-1β ↓ IL-18	[98]
Genipin	*Gardenia jasminoides*	CLP–induced sepsis ICR mice	1–5 mg/kg, i.v., immediately (0 h) or 0 and 24 h after CLP	TLR2/HMGB1/MyD88/TRIF/NF-κB (p65)/MAPK (p38/JNK/ERK)/IRF3; IκBα stabilization	↓ TNF-α ↓ IL-1β ↓ IL-6	[96]
Dioscin	*Dioscorea* *nipponica Makino*	Zymosan-induced SIRS C57BL/6J mice and Sprague Dawley rats	20–80 mg/kg for mice and 15–60 mg/kg for rats, orally, 7 days	TLR2/HMGB1/MyD88/NF-κb inhibition; IκBα stabilization	↓ TNF-α ↓ IL-1β ↓ IL-6 ↓ MPO ↓ MDA ↑ SOD ↓CD68^+^ macrophages	[97]

Legend: ↓, decreased; ↑, increased; AKT, protein kinase B; alpha-SMA, alpha-smooth muscle actin; CCl_4_, carbon tetrachloride; CLP, cecal ligation and puncture; col3a1, type III collagen a 1; COX-2, cyclooxygenase-2; cRaf, serine/threonine kinase in the Raf pathway; Egr1, early growth response1; ERK1/2, extracellular signal-regulated kinase 1/2; F4/80^+^, macrophage marker F4/80-positive cells; GCLC, glutamate-cysteine ligase catalytic subunit; GCLM, glutamate-cysteine ligase modifier subunit; HMGB1, high mobility group box 1; IFN-γ, interferon gamma; IL-1β, interleukin-1 beta; IL-4, interleukin-4; IL-6, interleukin-6; IL-10, interleukin-10; IL-18, interleukin-18; iNOS, inducible nitric oxide synthase; IκB, inhibitor of nuclear factor kappa B; IRAK1, interleukin-1 receptor-associated kinase 1; IRF3, interferon regulatory factor; JNK, c-Jun N-terminal kinase; MAPK, mitogen-activated protein kinase; MCP-1, monocyte chemoattractant protein-1; MCT, monocrotaline; MDA, malondialdehyde; MEK1/2, MAPK/ERK kinase 1/2; MKK3/6, MAPK kinase 3/6; MKK4, MAPK kinase 4; MMP9, matrix metalloproteinase 9; MPO, myeloperoxidase; MyD88, myeloid differentiation primary response 88; NF-κB, nuclear factor kappa B; NLRP3, Nucleotide-binding Oligomerization Domain-like Receptor Protein 3; NO, nitric oxide; Nrf2, nuclear factor erythroid 2–related factor 2; p38, p38 mitogen-activated protein kinase; LPS, lipopolysaccharide; PI3K, phosphoinositide 3-kinase; SIRS, Systemic inflammatory response syndrome; SOS, sinusoidal obstruction syndrome; TF, tissue factor; TLR2, toll-like receptor 2; TNF-α, tumor necrosis factor alpha; TRIF, TIR-domain-containing adapter-inducing interferon-β.

**Table 6:** Natural compounds targeting TLR9 signaling pathways in experimental models of DNA-sensing–driven inflammation and tissue injury.

Natural Compound	Sources	Experimental Model	Method of Administration	TLR9-Related Mechanism	Main Outcomes	Reference
Glycyrrhizin	*Glycyrrhiza glabra*	CLP-induced sepsis-associated ARDS C57BL/6 mice	15 mg/kg, i.p., single dose after CLP	TLR9/HMGB1/MyD88 inhibition	↓ Cit-H3 ↓ NETs in lung tissue ↓ IL-6	[103]
Curcumin	*Curcuma longa Linn*	Concanavalin A–induced autoimmune hepatitis BALB/c mice	200 mg/kg, orally, single dose 40 min before Concanavalin A injection	TLR9 inhibition	↓ TNF-α ↓ IFN-γ ↑ IL-10 ↓ Kupffer cell infiltration (F4/80^+^)	[72]
		CLP–induced AKI Sprague-Dawley rats	40 mg/kg, orally, single dose after CLP	TLR9/MYD88/IRF5/IRF7/NF-κB inhibition	↓ TNF-α ↓ IL-10 ↓ NGAL ↓ KIM-1 ↓ CysC	[102]
Tanshinone IIA	*Salvia miltiorrhiza Bunge*	Collagen–induced arthritis DBA/1 mice	5 mg/kg, orally, once a day from days 21 to 48 days	TLR9/RAGE/MMP9 inhibition	↓ TNF-α ↓ IL-1β ↓ IL-6	[104]
Oleanolic acid	*Olea europaea* *Salvia miltiorrhiza* *Sambucus chinensis*	STZ–induced diabetes Sprague Dawley rats	5 mg/kg, orally, 21 days	TLR-9/NF-κB inhibition	↓ IL-18 ↓ MDA	[105]
Oxymatrine	*Daphniphyllum* spp.	TNBS-induced colitis Sprague-Dawley rats	10–60 mg/kg, i.p., 7 days	TLR9/Myd88/NF-κB (p65) inhibition	↓ TNF-α ↓ IL-1β ↓ IL-6 ↓ IL-10 ↑ ZO-1 ↑ occludin ↑ claudin-2	[101]
Quercetin/Baicalein	Quercetin: *Allium cepa* *Vitis vinifera* *Solanum lycopersicum* *Brassica oleracea* Baicalein: *Lepisorus ussuriensis* *Scutellaria prostrata*	MCT-induced SOS Sprague Dawley rats	40 mg/kg, intragastrical administration, twice at 6 h and 30 h after MCT administration	TLR9/MyD88/NF-κB (p-p65/nuclear-p65/p-IκB)/Egr1/MAPK (p-ASK1/p-MEK1/2/p-cRaf/p-MKK3/6/p-MKK4/p38/JNK/ERK 1/2)/PI3K/AKT/mTOR inhibition; Nrf2 activation	↓ TNF-α ↓ IL-1β ↓ MDA ↓ MPO ↓ MMP-9 ↓ Serpine1 ↓ TF ↑ GCLC ↑ GCLM	[100]
Gastrodin	*Gastrodia elata*	Ischemic stroke injury (middle cerebral artery occlusion/reperfusion) Sprague Dawley rats	25–100 mg/kg, i.p., 3 days before surgery and an additional 7 days post-surgery.	mDNA/TLR9/JAK2/STAT3 inhibition	↓ TNF-α ↓ IL-1β ↓ IL-6 ↑ CAT ↑ GSH ↓ MDA ↑ SOD ↑ ATP ↑ T-ATPase	[106]
Betulinic acid	*Paeonia emodi* *Bowdichia virgilioides*	APAP-induced hepatotoxicity Sprague Dawley rats	25 mg/kg, orally, 15 days	TLR-9/NF-κB inhibition	↓ IL-18 ↓ MDA	[107]

Legend: ↓, decreased; ↑, increased; AKI, acute kidney injury; AKT, protein kinase B; ARDS, acute respiratory distress syndrome; ASK1, apoptosis signal-regulating kinase 1; ATP, adenosine triphosphate; CAT, catalase; CysC, cystatin-C; Cit-H3, citrullinated histone 3; CLP, Cecal ligation and puncture; cRaf, serine/threonine kinase in the Raf pathway; Egr1, early growth response1; ERK1/2, extracellular signal-regulated kinase 1/2; GCLC, glutamate-cysteine ligase catalytic subunit; GCLM, glutamate-cysteine ligase modifier subunit; GSH, glutathione; HMGB1, high mobility group box 1; IκB, inhibitor of nuclear factor kappa B; IFN-γ, interferon gamma; IL-6, interleukin-6; IL-10, interleukin-10; IL-18, interleukin-18; IRF5, interferon regulatory factor 5; IRF7, interferon regulatory factor 7; JNK, c-Jun N-terminal kinase; KIM-1, kidney injury molecule-1; MAPK, mitogen-activated protein kinase; MCT, monocrotaline; MDA, malondialdehyde; mDNA, mitochondrial DNA; MEK1/2, MAPK/ERK kinase 1/2; MKK3/6, MAPK kinase 3/6; MKK4, MAPK kinase 4; MMP9, matrix metalloproteinase 9; MPO, myeloperoxidase; MyD88, myeloid differentiation primary response 88; mTOR, mechanistic target of rapamycin; NETs, neutrophil extracellular traps; NF-κB, nuclear factor kappa B; NGAL, neutrophil gelatinase-associated lipocalin; Nrf2, nuclear factor erythroid 2–related factor 2; PI3K, phosphoinositide 3-kinase; RAGE, receptor for advanced glycation end product; SOD, superoxide dismutase; SOS, sinusoidal obstruction syndrome; STZ, streptozotocin; TF, tissue factor; TLR9, toll-like receptor 9; TNBS, 2,4,6-trinitrobenzene sulfonic acid; TNF-α, tumor necrosis factor alpha; ZO-1, zonula occluden.

**Table 7:** Overview of natural TLR modulators: chemical classification, physicochemical characteristics, and safety information [108].

Natural Compound	Chemical Class	Physicochemical Properties	Safety Data
Baicalin	Glycosyloxyflavone	MW = 446.4 g/mol; XLogP3 = 1.1; TPSA = 183 Å^2^; HBD = 6; HBA = 11	-
Paeoniflorin	Terpene glycoside	MW = 480.5 g/mol; XLogP3 = −1; TPSA = 164 Å^2^; HBD = 5; HBA = 11	Mouse (i.p.) LD_50_ = 3530 mg/kg; Mouse (i.v.) LD_50_ = 9530 mg/kg Adverse effects: sleep, somnolence.
Rotundic acid	Triterpenoid	MW = 488.7 g/mol; XLogP3 = 5.3; TPSA = 98 Å^2^; HBD = 4; HBA = 5	-
Ginsenoside Rb1	Ginsenoside	MW = 1109.3 g/mol; XLogP3 = 0.3; TPSA = 377 Å^2^; HBD = 15; HBA = 23	Mouse (i.p.) LD_50_ = 1110 mg/kg; Mouse (i.v.) LD_50_ = 243 mg/kg
Curcumin	Beta-diketone	MW = 368.4 g/mol; XLogP3 = 3.2; TPSA = 93.1 Å^2^; HBD = 2; HBA = 6	Mouse (i.p.) LD_50_ = 1500 mg/kg; Mouse (oral) LD_50_ > 2 g/kg; Rat (oral) LD_50_ > 5 g/kg; Rabbit (dermal) LD_50_ > 5 g/kg.
Quercetin	Pentahydroxyflavone	MW = 302.23 g/mol; XLogP3 = 1.5; TPSA = 127 Å^2^; HBD = 5; HBA = 7	Mouse (oral) LD_50_ = 159 mg/kg Mouse (i.p.) LD_50_ = 3 g/kg Mouse (s.c.) LD_50_ = 97 mg/kg Rat (oral) LD_50_ = 161 mg/kg. Adverse effects: somnolence, muscle weakness, respiratory depression;
Glycyrrhizin	Triterpenoid saponin	MW = 822.9 g/mol; XLogP3 = 3.7; TPSA = 267 Å^2^; HBD = 8; HBA = 16	Human (oral) TDLo = 5571 µg/kg/3D—changes in urine composition; Human (oral) TDLo = 280 mg/kg/4 weeks—somnolence; Human (oral) TDLo = 662 mg/kg/1 year—convulsions, muscle weakness; Rat (oral) LDLo = 3 g/kg; Rat (i.p.) LDLo = 2 g/kg; Mouse (oral) LD_50_ = 4320 mg/kg; Mouse (i.p.) LDLo = 1 g/kg; Mouse (i.v.) LD_50_ = 589 mg/kg—respiratory stimulation, other respiratory changes.
Resveratrol	Stilbene	MW = 228.24 g/mol; XLogP3 = 3.1; TPSA = 60.7 Å^2^; HBD = 3; HBA = 3	Rat (oral, 90 days, feed) BMDL_05_ = 344 mg/kg bw/day –body weight decrease; Human (oral, 1.5–3.0 g/day): mild, reversible ALT/AST increases
Salidroside	Glycoside	MW = 300.30 g/mol; XLogP3 = −0.6; TPSA = 120 Å^2^; HBD = 5; HBA = 7	Mouse (s.c.) LD_50_ = 28,600 µL/kg
Hesperetin	Trihydroxyflavanone	MW = 302.28 g/mol; XLogP3 = 2.4; TPSA = 96.2 Å^2^; HBD = 3; HBA = 6	-
Hesperidin	Flavanone disaccharide	MW = 610.6 g/mol; XLogP3 = –1.1; TPSA = 234 Å^2^; HBD = 8; HBA = 15	Mouse (i.p.) LD_50_ = 1 g/kg
Allicin	Sulfoxide	MW = 162.3 g/mol; XLogP3 = 1.3; TPSA = 61.6 Å^2^; HBD = 0; HBA = 3	Mouse (s.c.) LD_50_ = 120 mg/kg; Mouse (i.v.) LD_50_ = 60 mg/kg
Magnoflorine	Alkaloid	MW = 342.4 g/mol; XLogP3 = 2.7; TPSA = 58.9 Å^2^; HBD = 2; HBA = 4	Mouse (i.p.) LD_50_ = 19,600 µg/kg; Mouse (s.c.) LD_50_ = 138 mg/kg; Mouse (i.v.) LD_50_ = 20 mg/kg
Magnolol	Biphenyl	MW = 266.3 g/mol; XLogP_3_ = 5; TPSA = 40.5 Å^2^; HBD = 2; HBA = 2	Mouse (oral) LD_50_ = 2200 mg/kg
Procyanidin	Proanthocyanidin oligomer	MW = 594.5 g/mol; XLogP3 = 2; TPSA = 230 Å^2^; HBD = 10; HBA = 13	-
Chlorogenic acid	Cinnamate ester	MW = 354.31 g/mol; XLogP3 = −0.4; TPSA = 165 Å^2^; HBD = 6; HBA = 9	Rat (i.p.) LDLo = 4 g/kg
Paeonol	Phenol	MW = 166.17 g/mol; XLogP3 = 2; TPSA = 46.5 Å^2^; HBD = 1; HBA = 3	Mouse (oral) LD_50_ = 490 mg/kg; Mouse (i.p.) LD_50_ = 781 mg/kg—altered sleep time; Mouse (i.v.) LD_50_ = 196 mg/kg—altered sleep time
Anthocyanins	Flavonoid	MW = 207.25 g/mol; TPSA = 1 Å^2^; HBD = 0; HBA = 0	-
*Berberine*	Alkaloid	MW = 336.4 g/mol; XLogP3 = 3.6; TPSA = 40.8 Å^2^; HBD = 0; HBA = 4	Rat (i.p.) LD > 500 mg/kg; Mouse (oral) LD_50_ = 329 mg/kg; Mouse (s.c.) LD_50_ = 18 mg/kg; Rabbit (s.c.) LDLo = 100 mg/kg.
Genipin	Iridoid monoterpenoid	MW = 226.23 g/mol; XLogP3 = −0.7; TPSA = 76 Å^2^; HBD = 2; HBA = 5	Mouse (oral) LD_50_ = 237 mg/kg; Mouse (i.p.) LD_50_ = 190 mg/kg; Mouse (i.v.) LD_50_ = 153 mg/kg.
Baicalein	Trihydroxyflavone	MW = 270.24 g/mol; XLogP3 = 1.7; TPSA = 87 Å^2^; HBD = 3; HBA = 5	-
Dioscin	Spirostanyl glycoside	MW = 869.0 g/mol; XLogP3 = 1.3; TPSA = 236 Å^2^; HBD = 8; HBA = 16	Mouse (s.c.) LD_50_ > 300 mg/kg
Tanshinone IIA	Diterpenoid	MW = 294.3 g/mol; XLogP3 = 4.3; TPSA = 47.3 Å^2^; HBD = 0; HBA = 3	-
Oleanolic acid	Triterpenoid	MW = 456.7 g/mol; XLogP3 = 7.5; TPSA = 57.5 Å^2^; HBD = 2; HBA = 3	Rat (oral) LD_50_ > 2 g/kg; Rat (i.p.) LD_50_ > 2 g/kg; Mouse (oral) LD_50_ > 2 g/kg; Mouse (i.p.) LD_50_ = 1500 mg/kg
Oxymatrine	Alkaloid	MW = 264.36 g/mol; XLogP3 = 1; TPSA = 38.4 Å^2^; HBD = 0; HBA = 2	Mouse (i.p.) LD_50_ = 521 mg/kg; Mouse (i.m.) LD_50_ = 257 mg/kg; Mouse (i.v.) LD_50_ = 150 mg/kg
Gastrodin	Phenolic glycoside	MW = 286.28 g/mol; XLogP3 = −0.8; TPSA = 120 Å^2^; HBD = 5; HBA = 7	-
Betulinic acid	Triterpenoid	MW = 456.7 g/mol; XLogP3 = 8.2; TPSA = 57.5 Å^2^; HBD = 2; HBA = 3	-

Legend: ALT, alanine aminotransferase; AST, aspartate aminotransferase; BMDL_05_, benchmark dose lower confidence limit for 5% response; bw, body weight; HBA, hydrogen bond acceptors; HBD, hydrogen bond donors; i.m., intramuscular; i.p., intraperitoneal; i.v., intravenous; LDLo, lowest published lethal dose; LD_50_, median lethal dose; mg/kg, milligrams per kilogram; MW, molecular weight; s.c., subcutaneous; TDLo, lowest published toxic dose; TPSA, topological polar surface area; µg/kg, micrograms per kilogram; µL/kg, microlitres per kilogram; XLogP_3_, computed octanol–water partition coefficient. Chemical classification, physicochemical characteristics, and safety data were obtained from the PubChem database (National Center for Biotechnology Information) [108].

**Table 8:** Representative examples of natural compounds showing neutral or context-dependent effects on TLR signaling.

Compound	Model/Cell	TLR Target	Agonist/Context	Effect vs. Control
Chlorogenic acid	Primary rat hepatic stellate cells	TLR4/MyD88	LPS	No change in TLR4 or MyD88 expression vs. control; downstream NF-κB/ROS inhibited [113]
Glycyrrhizin	RAW264.7 macrophages and airway epithelium models	TLR4/CD14	LPS or HMGB1-rich supernatants	No effect on TLR4/CD14 expression; reduces TLR4 translocation to lipid rafts and downstream signaling [114]
Resveratrol	RAW264.7 macrophages	TLR2/3/4/9 pathways	Poly I:C (TLR3), LPS (TLR4), Pam3CSK4 (TLR2), CpG (TLR9)	Inhibits TRIF-dependent (TLR3/4) NF-κB, no inhibition on MyD88-dependent TLR2/9 [115]
Berberine	LPS-stimulated macrophages	TLR4/MyD88	LPS	TLR4 protein unchanged; interferes with TLR4–MyD88 signaling [116]
Curcumin	Hepatocytes/marrow (ischemia–reperfusion in vivo and in vitro)	TLR4/NF-κB	H/R injury or LPS	No significant change vs. control in unstimulated groups; inhibits TLR4/NF-κB only under injurious stimulation [117]

Legend: CD14, Cluster of differentiation 14 (co-receptor for TLR4); CpG, Cytosine–phosphate–guanine oligodeoxynucleotides; HMGB1, High-mobility group box 1; H/R, Hypoxia/reoxygenation; LPS, Lipopolysaccharide; MyD88, Myeloid differentiation primary response 88 (TLR adaptor protein); NF-κB, Nuclear factor kappa-light-chain-enhancer of activated B cells; Pam3CSK4, Synthetic triacylated lipopeptide; Poly I:C, Polyinosinic–polycytidylic acid; ROS, Reactive oxygen species; TLR, Toll-like receptor; TRIF, TIR-domain-containing adapter-inducing interferon-β.

## Data Availability

No new data were created or analyzed in this study. Data sharing is not applicable to this article.
